# Functional identification of complex cells from spike times and the decoding of visual stimuli

**DOI:** 10.1186/1471-2202-16-S1-P300

**Published:** 2015-12-18

**Authors:** Aurel A Lazar, Nikul H Ukani, Yiyin Zhou

**Affiliations:** 1 Department of Electrical Engineering, Columbia University, New York, NY 10027, USA

## 

Neural circuits built with complex cells play a key role in modeling the primary visual cortex. The encoding capability of an ensemble of complex cells has not been systematically studied, however. Can visual scenes be reconstructed using the spike times generated by an ensemble of complex cells? Can the processing taking place in complex cells be identified with high accuracy? Processing by complex cells has the complexity of Volterra models [1]. General Volterra based models call, among others, for efficient functional identification and decoding algorithms.

We demonstrate that complex cells exhibit Volterra dendritic stimulus processors (Volterra DSPs) that are analytically and computationally tractable. Decoding and identification problems arising in neural circuits built with complex cells can be efficiently solved as rank minimization problems [2]. We provide (i) an algorithm that reconstructs the visual stimuli based on the spike times generated by circuits with widely employed complex cells models (Complex Cell Time Decoding Machines), and (ii) propose a mechanistic algorithm for functionally identifying the processing in complex cells using the spike times they generate (Complex Cell Channel Identification Machines). These algorithms are based on the key observation that the functional identification of processing in a single complex cell is dual to the problem of decoding stimuli encoded by an ensemble of complex cells.

**Figure 1:**
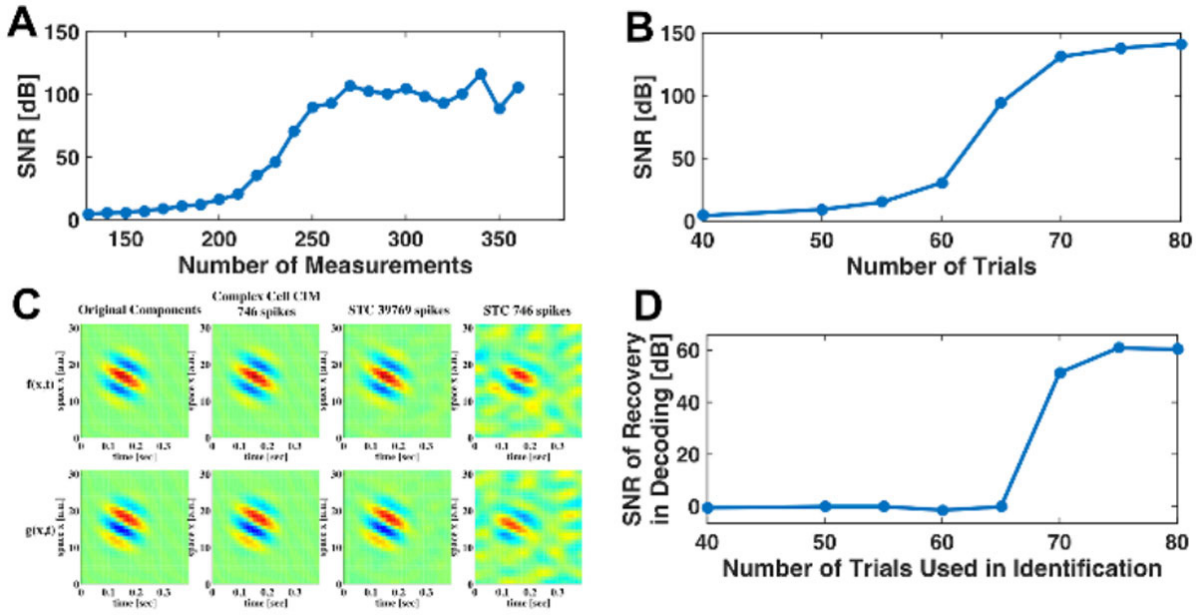
**A. SNR of reconstruction when varying the number of measurements/spikes (dimension of the input space: 117)**. B. Mean SNR of identified complex cell DSP when varying the number of input trials used in identification. C. Comparison of identification by Complex Cell CIM with STC. Quadrature pair Gabor filters (1st column) identified with Complex Cell CIM with 746 spikes (2nd column, SNR: 123.08 [dB], 88.93 [dB]), and with STC using 39, 769 spikes (3rd column, SNR: 16.77 [dB], 17.90 [dB]), and using 746 spikes (4th column, SNR: 0.21 [dB], 0.52 [dB]). D. Evaluating identification quality in the input space. SNR of reconstruction of novel stimuli assumed to be encoded with the identified DSPs.

In addition, we show that the number of spikes needed for perfect reconstruction of a band-limited stimulus is proportional to the dimension of the stimulus space rather than the square of its dimension, thereby reducing the required number of neurons/measurements to a physiologically plausible range. This result demonstrates that visual stimuli can be efficiently reconstructed from the amplitude information carried in the complex cells alone. Similar results obtained for identification establish the computational tractability of higher order Volterra DSPs. We provide examples of perfect reconstruction of space-time stimuli (Figure 1A) and examples of identification of complex cell DSPs (Figure 1B). We demonstrate that our identification algorithms substantially outperform algorithms based on spike-triggered covariance (STC) (Figure 1C). Finally, we evaluate our identification algorithms by reconstructing novel stimuli in the input space using identified Volterra DSPs (Figure 1D) [3].
